# Supplementary material for: Pre-marking chromatin with H3K4 methylation is required for accurate zygotic genome activation and development
Source: Nat Commun. 2025 Dec 19;16:11334. doi: 10.1038/s41467-025-67692-7 (PMC12722762; doi:10.1038/s41467-025-67692-7)
Supplement: Supplementary file 1 — Supplementary Infomation [file 41467_2025_67692_MOESM1_ESM.pdf]

Fig S1.

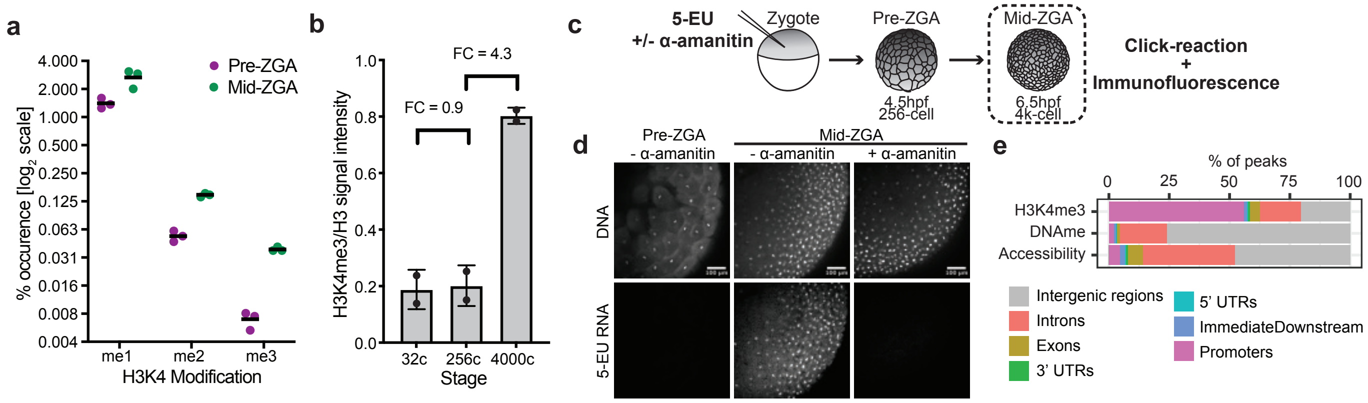

a
Pre-ZGA
Mid-ZGA
% occurence [log2 scale]
4.000
2.000
1.000
0.500
0.250
0.125
0.063
0.031
0.016
0.008
0.004
me1
me2
me3
H3K4 Modification
b
H3K4me3/H3 signal intensity
FC = 0.9
FC = 4.3
1.0
0.8
0.6
0.4
0.2
0.0
32c
256c
4000c
Stage
c
5-EU
+/- α-amanitin
Zygote
Pre-ZGA
4.5hpf
256-cell
Mid-ZGA
6.5hpf
4k-cell
Click-reaction
+
Immunofluorescence
d
Pre-ZGA
- α-amanitin
Mid-ZGA
- α-amanitin
+ α-amanitin
DNA
5-EU RNA
e
% of peaks
0
25
50
75
100
H3K4me3
DNAme
Accessibility
Intergenic regions
Introns
Exons
3' UTRs
5' UTRs
ImmediateDownstream
Promoters

SUPPLEMENTARY INFORMATION

Figure S1. Profiling histone modifications and epigenetic factors present on pre-ZGA embryo chromatin.

(a) Percent abundance of modifications of H3K4 peptide at pre-ZGA and ZGA stages represented on a log_2 scale. (b) Quantification of H3K4me3 signal intensity relative to H3 as seen in the Western blot in Fig.1c at 32-cell, 256-cell (pre-ZGA) and 4000-cell (mid-ZGA) stages. Fold change is calculated relative to 256-cell stage. (c) Illustration of 5-EU labeling of nascent RNA in early embryos for immunofluorescence. (d) Representative images demonstrating inhibition of transcription α-amanitin-treated embryos. Nascent transcripts are labeled using 5-EU (bottom) and DNA is labeled using Sir-DNA (top). Scale bars: 100µm. (e) Genomic distribution of called peaks for H3K4me3 ChIP sequencing, MBD sequencing and CATaDa on pre-ZGA embryos (4.5hpf).

Fig S2.

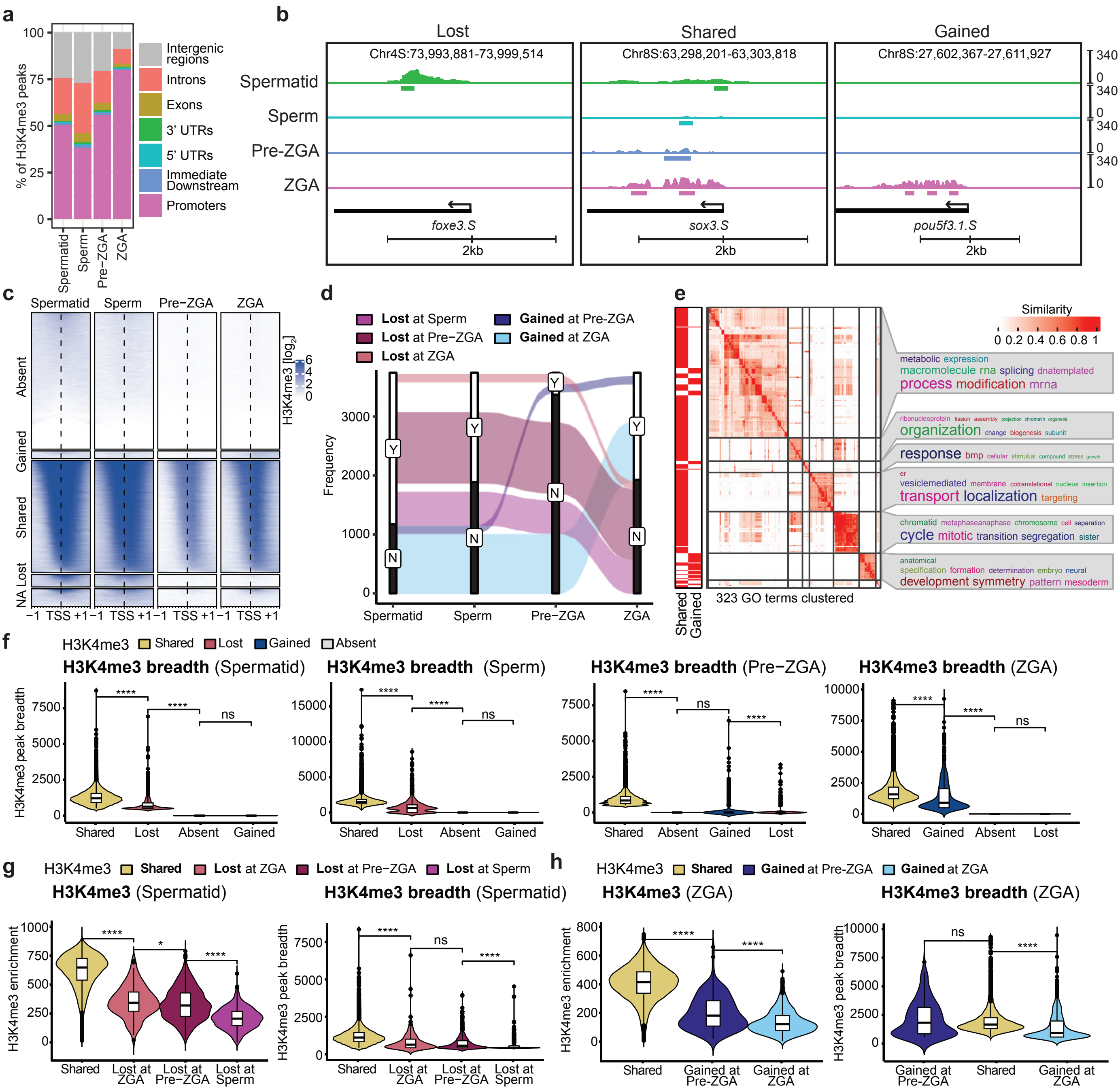

a
b
c
d
e
f
g
h
% of H3K4me3 peaks
Intergenic regions
Introns
Exons
3' UTRs
5' UTRs
Immediate Downstream
Promoters
Spermatid
Sperm
Pre-ZGA
ZGA
Lost
Shared
Gained
Chr4S:73,993,881-73,999,514
Chr8S:63,298,201-63,303,818
Chr8S:27,602,367-27,611,927
foxe3.S
sox3.S
pou5f3.1.S
2kb
340
0
Absent
Gained
Shared
NA Lost
−1 TSS +1
H3K4me3 [log2]
Lost at Sperm
Lost at Pre−ZGA
Lost at ZGA
Gained at Pre-ZGA
Gained at ZGA
Frequency
Y
N
Similarity
323 GO terms clustered
metabolic expression macromolecule rna splicing dnatemplated process modification mrna
ribonucleoprotein fission assembly projection chromatin organelle organization change biogenesis subunit
response bmp cellular stimulus compound stress growth
er vesiclemediated membrane cotranslational nucleus insertion transport localization targeting
chromatid metaphaseanaphase chromosome cell separation cycle mitotic transition segregation sister
anatomical specification formation determination embryo neural development symmetry pattern mesoderm
H3K4me3 Shared Lost Gained Absent
H3K4me3 breadth (Spermatid)
H3K4me3 breadth (Sperm)
H3K4me3 breadth (Pre−ZGA)
H3K4me3 breadth (ZGA)
H3K4me3 peak breadth
****
ns
H3K4me3 Shared Lost at ZGA Lost at Pre−ZGA Lost at Sperm
H3K4me3 (Spermatid)
H3K4me3 enrichment
H3K4me3 Shared Gained at Pre-ZGA Gained at ZGA
H3K4me3 (ZGA)
*

Figure S2. H3K4me3 peak intensity closely correlates with the dynamics of H3K4me3 localization.

(a) Genomic distribution of H3K4me3 peaks at all time points. (b) IGV tracks of genes representing LOST, SHARED and GAINED H3K4me3 dynamics groups. (c) Heatmap of H3K4me3 intensity (log_2) around TSS (+/- 1 kb) at spermatid, sperm, pre-ZGA and ZGA time points clustered by H3K4me3 dynamics groups. (d) Alluvial plot illustrating dynamics of promoter H3K4me3 for the GAINED and LOST groups. (e) GO analysis for the SHARED and GAINED H3K4me3 dynamics groups that clusters significantly enriched GO terms (adj. p-value < 0.01) based on their similarity. For the LOST group, no GO term is significantly enriched. (f) Distributions of H3K4me3 peak breadth at promoters in each H3K4me3 dynamics group (bp) at each time point - spermatid, sperm, pre-ZGA, post-ZGA respectively. Statistical test: one-sided Wilcoxon rank-sum test with alternative hypothesis that values of left distribution tend to be larger than right distribution; p-values: (****)<=0.0001, (***)<=0.001, (**) <= 0.01, (*)<=0.05; n.s. are p-values > 0.05. (g) Distribution of H3K4me3 promoter enrichment (left) and H3K4me3 peak breadth (bp) (right) at promoters for groups in spermatid. (h) Distribution of H3K4me3 promoter enrichment (left) and H3K4me3 peak breadth (bp) (right) at promoters for groups in post-ZGA.

Fig S3.

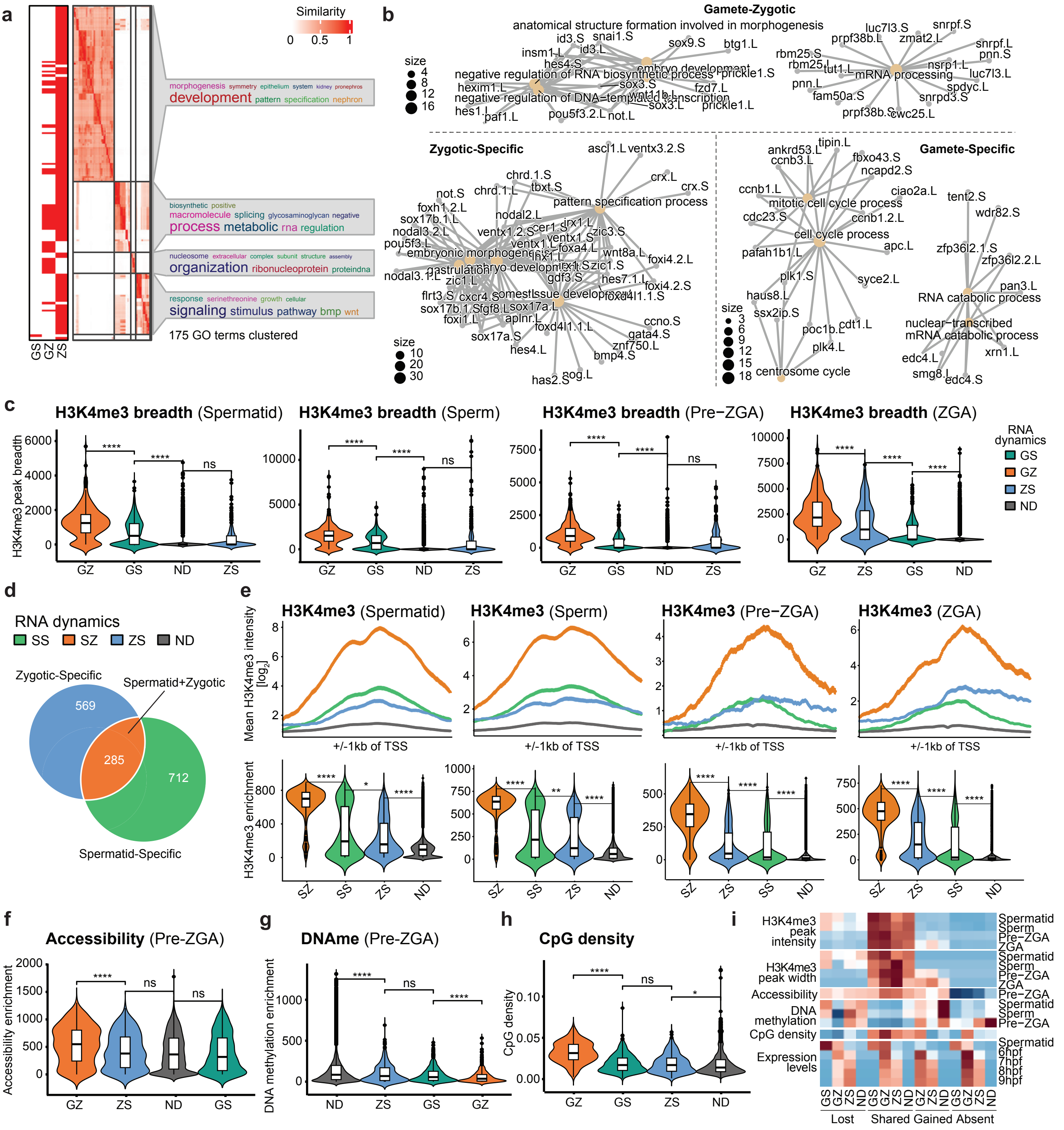

a
Similarity
0 0.5 1
morphogenesis symmetry epithelium system kidney pronephros development pattern specification nephron
biosynthetic positive macromolecule splicing glycosaminoglycan negative process metabolic rna regulation
nucleosome extracellular complex subunit structure assembly organization ribonucleoprotein proteindna
response serinethreonine growth cellular signaling stimulus pathway bmp wnt
175 GO terms clustered
GS GZ ZS
b
Gamete-Zygotic
Zygotic-Specific
Gamete-Specific
anatomical structure formation involved in morphogenesis
negative regulation of RNA biosynthetic process
negative regulation of DNA-templated transcription
embryo development
mRNA processing
pattern specification process
embryonic morphogenesis
gastrulation
embryo development
tissue development
mitotic cell cycle process
cell cycle process
centrosome cycle
RNA catabolic process
nuclear-transcribed mRNA catabolic process
c
H3K4me3 breadth (Spermatid)
H3K4me3 breadth (Sperm)
H3K4me3 breadth (Pre−ZGA)
H3K4me3 breadth (ZGA)
H3K4me3 peak breadth
RNA dynamics
GS
GZ
ZS
ND
d
RNA dynamics
SS SZ ZS ND
Zygotic-Specific
Spermatid+Zygotic
569
285
712
Spermatid-Specific
e
H3K4me3 (Spermatid)
H3K4me3 (Sperm)
H3K4me3 (Pre−ZGA)
H3K4me3 (ZGA)
Mean H3K4me3 intensity [log2]
+/-1kb of TSS
H3K4me3 enrichment
f
Accessibility (Pre-ZGA)
Accessibility enrichment
g
DNAme (Pre-ZGA)
DNA methylation enrichment
h
CpG density
CpG density
i
H3K4me3 peak intensity
H3K4me3 peak width
Accessibility
DNA methylation
CpG density
Expression levels
Spermatid
Sperm
Pre-ZGA
ZGA
6hpf
7hpf
8hpf
9hpf
Lost
Shared
Gained
Absent

Figure S3. H3K4me3 marks gene promoters independently of transcription between two transcriptionally active time points.

(a) GO analysis for the different RNA dynamics groups that clusters significantly enriched GO terms (adj. p-value < 0.01) based on their similarity. (b) Networks showing enriched GO terms, their relation and associated genes for (top) Gamete-Zygotic (GZ) genes, (bottom left) Zygotic-Specific (ZS) genes and (bottom right) Gamete-Specific (GS) genes. (c) Distributions of H3K4me3 peak breadth (bp) at promoters for the RNA dynamics groups at each time point - spermatid, sperm, pre-ZGA, post-ZGA. Statistical test: one-sided Wilcoxon rank-sum test with alternative hypothesis that values of left distribution tend to be larger than right distribution; p-values: (****)<=0.0001, (***)<=0.001, (**) <= 0.01, (*)<=0.05; n.s. are p-values > 0.05. (d) Venn diagram denoting overlap of genes detected in the spermatid (total RNA) and at ZGA stages (nascent). Colors are based on the dynamics of RNA expression across stages. (e) (Top) H3K4me3 around TSS (+/-1kb) for expression dynamics groups at each timepoint - spermatid, sperm, pre-ZGA, post-ZGA respectively. (Bottom) H3K4me3 promoter enrichment in each group for each timepoint. Statistical test: one-sided Wilcoxon rank-sum test with alternative hypothesis that values of left distribution tend to be larger than right distribution; p-values: (****)<=0.0001, (***)<=0.001, (**) <= 0.01, (*)<=0.05; n.s. are p-values > 0.05 (f) Accessibility promoter enrichment around TSS (+/-1kb) in each RNA dynamics group for pre-ZGA stage. (g) DNA methylation promoter enrichment around TSS (+/-1kb) in each RNA dynamics group for pre-ZGA stage. (h) Comparison of promoter CpG density around TSS (+/-1kb) in each RNA dynamics group for pre-ZGA stage. (i) Heatmap showing the z-scored mean of H3K4me3 enrichment and peak width, accessibility, CpG density and DNA methylation at promoters and expression levels for available timepoints for the intersections of major H3K4me3 dynamics groups with RNA dynamics groups.

Fig S4.

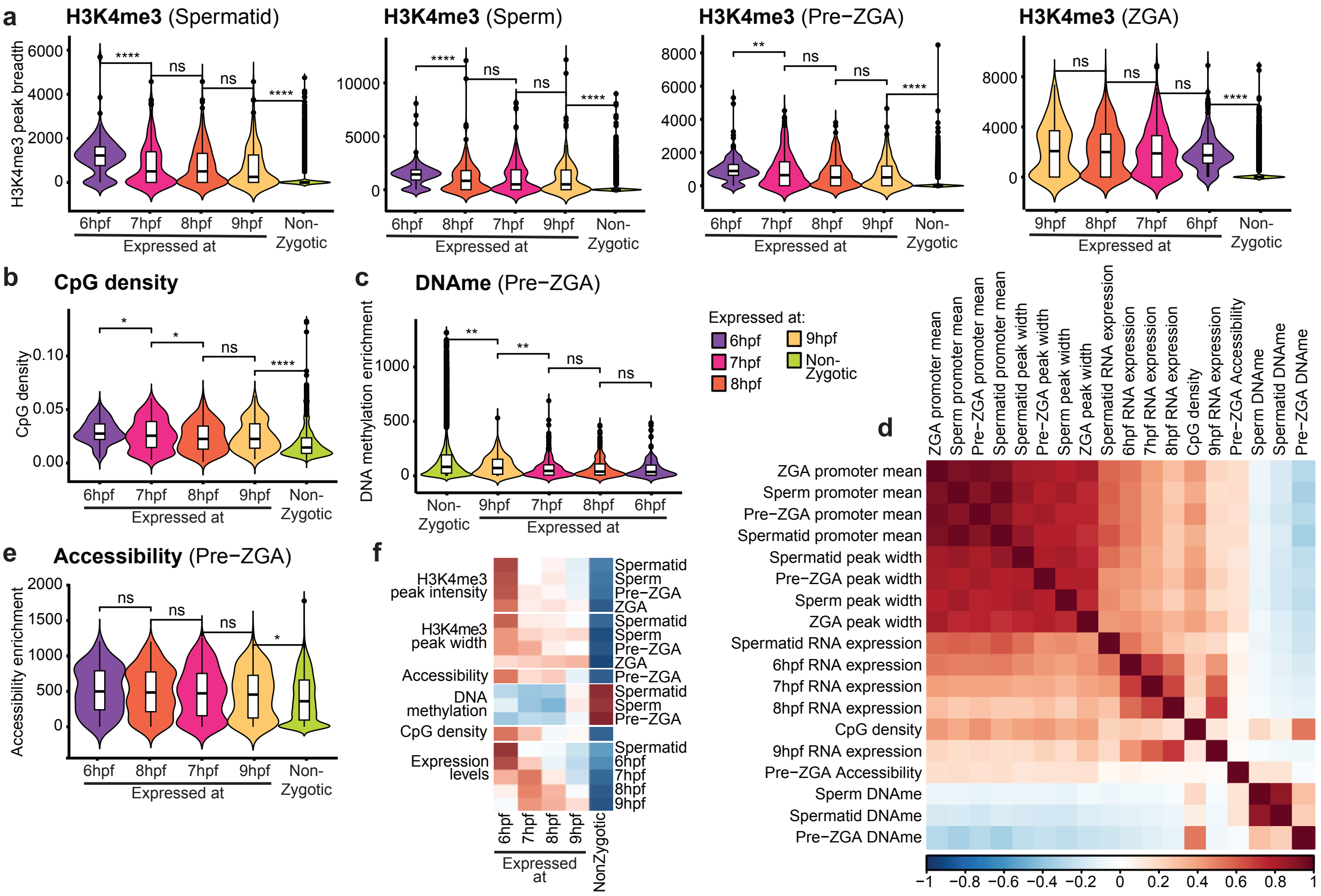

a
H3K4me3 (Spermatid)
H3K4me3 (Sperm)
H3K4me3 (Pre−ZGA)
H3K4me3 (ZGA)
H3K4me3 peak breadth
6hpf 7hpf 8hpf 9hpf Non-Zygotic
Expressed at
b
CpG density
c
DNAme (Pre−ZGA)
DNA methylation enrichment
Expressed at:
6hpf 7hpf 8hpf 9hpf Non-Zygotic
d
ZGA promoter mean
Sperm promoter mean
Pre−ZGA promoter mean
Spermatid promoter mean
Spermatid peak width
Pre−ZGA peak width
Sperm peak width
ZGA peak width
Spermatid RNA expression
6hpf RNA expression
7hpf RNA expression
8hpf RNA expression
CpG density
9hpf RNA expression
Pre−ZGA Accessibility
Sperm DNAme
Spermatid DNAme
Pre−ZGA DNAme
−1 −0.8 −0.6 −0.4 −0.2 0 0.2 0.4 0.6 0.8 1
e
Accessibility (Pre−ZGA)
Accessibility enrichment
f
H3K4me3 peak intensity
H3K4me3 peak width
Accessibility
DNA methylation
CpG density
Expression levels
Spermatid
Sperm
Pre−ZGA
ZGA
6hpf 7hpf 8hpf 9hpf
Expressed at
NonZygotic

Figure S4. Genes pre-marked by H3K4me3 are expressed early during zygotic genome activation.
(a) Distributions of H3K4me3 peak breadth (bp) at promoters for the ZGA timing groups at each time point - spermatid, sperm, pre-ZGA, post-ZGA respectively. (b) Comparison of promoter CpG density around TSS (+/-1kb) in each expression timing group for pre-ZGA stage. (c) DNA methylation promoter enrichment around TSS (+/-1kb) in each expression timing group for pre-ZGA stage. (d) Accessibility promoter enrichment around TSS (+/-1kb) in each expression timing group for pre-ZGA stage. (e) Heatmap showing the z-scored mean of H3K4me3 enrichment and peak width, accessibility, CpG density, DNA methylation, H3K4me3 enrichment and peak width at promoters and expression levels for available timepoints for genes grouped by expression timing. (f) Pairwise linear correlation computed between all presented data modalities.

Fig S5.

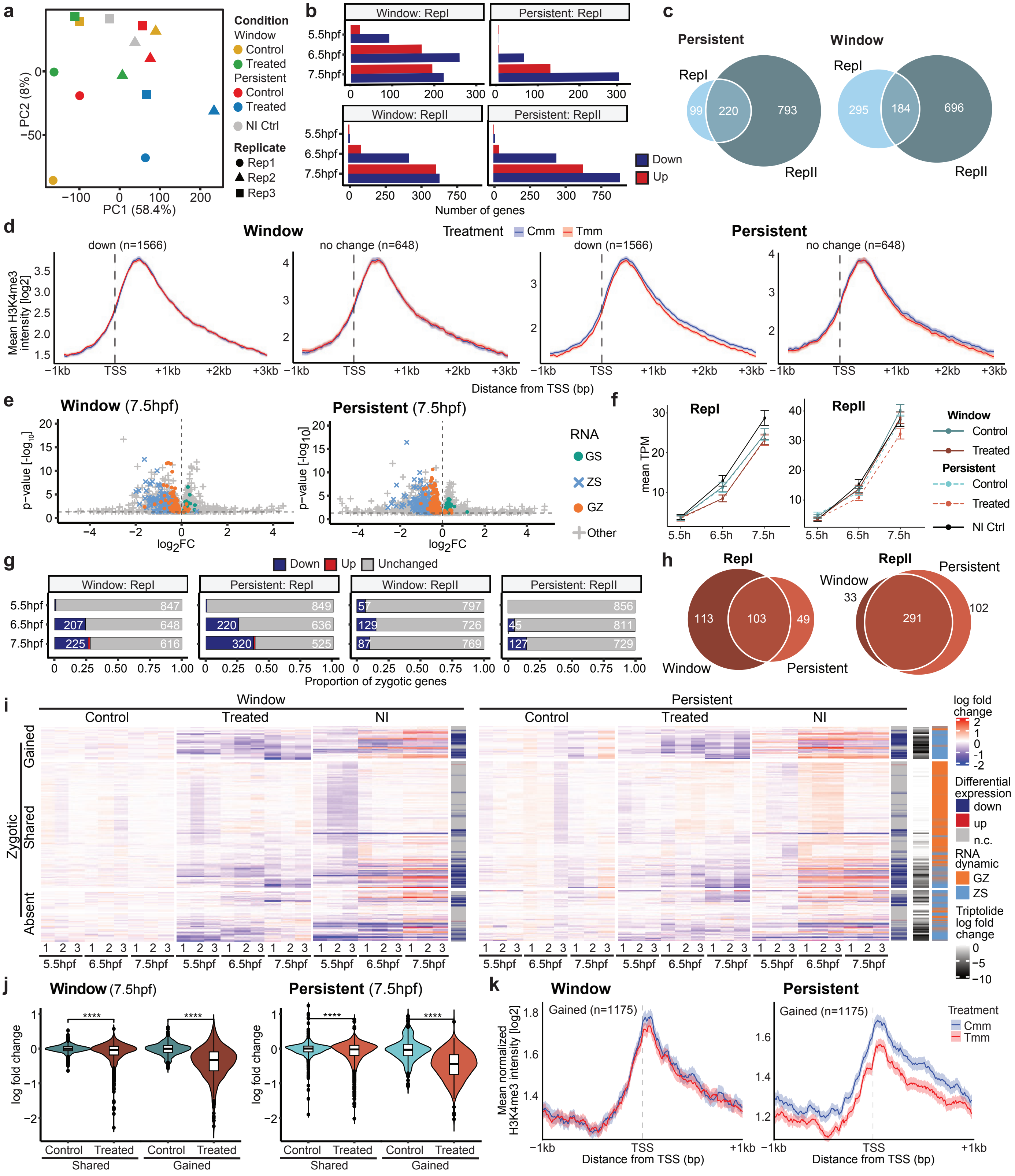

a
Condition
Window
Control
Treated
Persistent
Control
Treated
NI Ctrl
Replicate
Rep1
Rep2
Rep3
PC1 (58.4%)
PC2 (8%)
b
Window: RepI
Persistent: RepI
Window: RepII
Persistent: RepII
5.5hpf
6.5hpf
7.5hpf
Down
Up
Number of genes
c
Persistent
Window
RepI
RepII
99
220
793
295
184
696
d
Window
Persistent
Treatment
Cmm
Tmm
down (n=1566)
no change (n=648)
Mean H3K4me3 intensity [log2]
Distance from TSS (bp)
e
Window (7.5hpf)
Persistent (7.5hpf)
RNA
GS
ZS
GZ
Other
p−value [-log10]
log2FC
f
RepI
RepII
mean TPM
Window
Control
Treated
Persistent
Control
Treated
NI Ctrl
g
Down
Up
Unchanged
Window: RepI
Persistent: RepI
Window: RepII
Persistent: RepII
847
207
648
225
616
849
220
636
320
525
57
797
129
726
87
769
856
45
811
127
729
Proportion of zygotic genes
h
RepI
RepII
Window
Persistent
113
103
49
33
291
102
i
Window
Persistent
Control
Treated
NI
Zygotic
Gained
Shared
Absent
log fold change
Differential expression
down
up
n.c.
RNA dynamic
GZ
ZS
Triptolide log fold change
j
Window (7.5hpf)
Persistent (7.5hpf)
log fold change
Control
Treated
Shared
Gained
****
k
Window
Persistent
Gained (n=1175)
Treatment
Cmm
Tmm
Mean normalized H3K4me3 intensity [log2]
Distance from TSS (bp)

Figure S5. Promoter H3K4me3 during early embryonic cell divisions is required for proper ZGA.

(a) Principal component analysis comparing H3K4me3 counts within a genome-wide consensus peak set defined as the intersection of peaks called in at least 2 out of 3 biological replicates in wildtype samples. (b) Counts of differentially expressed genes at 5.5hpf, 6.5hpf and 7.5hpf time points in window and persistent conditions compared to their respective controls for both biological replicates (adj. p-value < 0.05). (c) Overlap of downregulated genes across all three timepoints between biological replicates in window (left) and persistent (right) depletion conditions compared to their respective controls. (d) Meta plots depicting H3K4me3 signal from the promoters (TSS -1 kb) into the gene body (TSS +3 kb) of genes whose expression was either downregulated or did not significantly change at 6.5 hpf in the window (left) or persistent treatment (right) (union of DEGs in two replicates of RNA-seq experiments). P-values were calculated using a Kolmogorov-Smirnov test: p-value=n.s. for window-treatment comparisons; For persistent treatment: downregulated genes p-value=0.02, no change genes p-value=n.s. (e) Volcano plots displaying expression fold change and adj. p-value (cutoff: 0.05) in treated condition compared to control condition at 7.5 hpf colored by RNA dynamics groups for (left) window depletion and (right) persistent depletion for biological replicate 2. Density plots on top represent the distributions of $\log_2$ fold change for the RNA dynamics group. (f) Mean TPM values of all Zygotic-Specific (ZS) genes in treated and control embryos under window and persistent depletion at 5.5hpf, 6.5hpf and 7.5hpf, shown for biological replicate 1 (left) and replicate 2 (right). Error bars visualize the standard error across technical replicates. (g) Number of differentially expressed and unchanged zygotic genes at 5.5hpf, 6.5hpf and 7.5hpf time points in window and persistent treatment conditions compared to their controls in biological replicate 1 (left) and replicate 2 (right) (adj. p-value < 0.05). *Zygotic genes = Gamete+Zygotic (GZ) + Zygotic-Specific (ZS).* (h) Overlap of downregulated genes across all three timepoints between window and persistent depletion conditions compared to their control conditions in biological replicate 1 (left) and 2 (right). (i) Expression of zygotic genes in three technical replicates per sample, clustered by H3K4me3 dynamics group (SHARED, GAINED, and ABSENT) for biological replicate 2. Log_2 fold changes are calculated relative to the mean TPM of the corresponding control condition at each timepoint. Genes are annotated with differential expression status separately for the window and persistent depletion conditions, as well as by RNA dynamics group. A column on the right displays $\log_2$ fold change values under transcriptional inhibition using triptolide at 7 hpf (adapted from Phelps et al., 2022), representing the maximum transcriptional downregulation expected for each gene at this stage. (j) Log_2 fold change of gene expression levels of all zygotic genes in SHARED and GAINED groups calculated over mean TPM of respective control technical replicates in window (left) and persistent (right) depletion conditions at 7.5 hpf in biological replicate 2. Statistical test: one-sided Wilcoxon rank-sum test with alternative hypothesis that values of left distribution tend to be larger than right distribution; p-values: (****)<=0.0001, (***)<=0.001, (**) <= 0.01, (*)<=0.05; n.s. are p-values > 0.05. (k) Meta plots depicting H3K4me3 signal at the promoters (TSS +/-1 kb) of genes from the Gained group at 6.5 hpf in the window (left) or persistent treatment (right) (union of DEGs in two replicates of RNA-seq experiments). P-values were calculated using a Kolmogorov-Smirnov test: p-value < 0.05 for window-treatment comparisons; p < 0.05 for persistent treatment comparisons.

Fig S6.

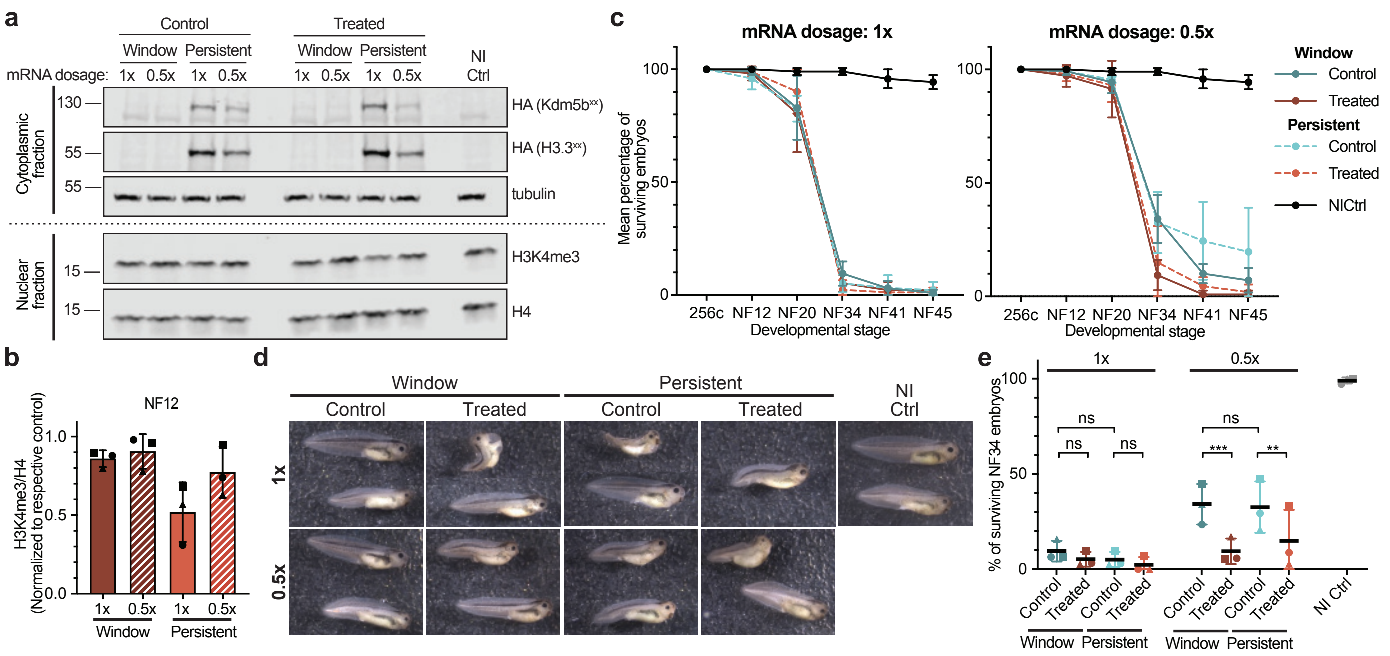

a
Control
Treated
Window
Persistent
mRNA dosage: 1x 0.5x 1x 0.5x
NI Ctrl
Cytoplasmic fraction
Nuclear fraction
HA (Kdm5b^xx)
HA (H3.3^xx)
tubulin
H3K4me3
H4
b
NF12
H3K4me3/H4 (Normalized to respective control)
c
mRNA dosage: 1x
mRNA dosage: 0.5x
Mean percentage of surviving embryos
Developmental stage
256c NF12 NF20 NF34 NF41 NF45
Window
Control
Treated
Persistent
NICtrl
d
1x
0.5x
e
% of surviving NF34 embryos
ns
***
**
NI Ctrl

Figure S6. Pre-ZGA H3K4 methylation is essential for embryonic viability and correct zygotic gene expression.

(a) (Top) Western blot of cytoplasmic fractions from NF12 embryos showing successful translation of ectopic HA-tagged $Kdm5b^{wt}$ + $H3.3^{K4M}$ (treatment) or $Kdm5b^{ci}$ + $H3.3^{wt}$ (control) mRNA in persistent depletion conditions and their corresponding degradation following auxin (IAA) induction in window depletion conditions. Tubulin serves as a loading control. "NI" denotes non-injected controls. (Bottom) Western blot of nuclear fractions of corresponding NF12 embryos showing H3K4me3 signal with H4 serving as a loading control in treated, control and NI conditions. (b) Quantification of H3K4me3 intensity normalized to H4 intensity in treated embryos relative to their respective controls in window and persistent depletion conditions. Replicates are denoted by different point styles. (c) Survival line plot of treated and control embryos injected with full (1x, left) and half (0.5x, right) mRNA dosage in window and persistent depletion conditions, alongside non-injected controls. Survival was assessed at key developmental timepoints (X-axis). The Y-axis represents the mean percentage of pre-ZGA embryos that survived to each respective stage. Data represent n=3 independent experiments, N>35 embryos per condition; error bars indicate standard deviation. (d) Representative phenotype images of embryos for each condition at NF41. (e) Column scatter plot showing the weighted mean percentage of surviving embryos at NF34 for all the above-described conditions. Independent biological replicates are represented with different point styles (n=3). Error bars indicate standard deviation. Asterisks denote significance from Fisher's exact test (one-tailed) performed on pooled embryo count data for each condition (alive vs dead embryos). **P-value < 0.01.

Fig S7.

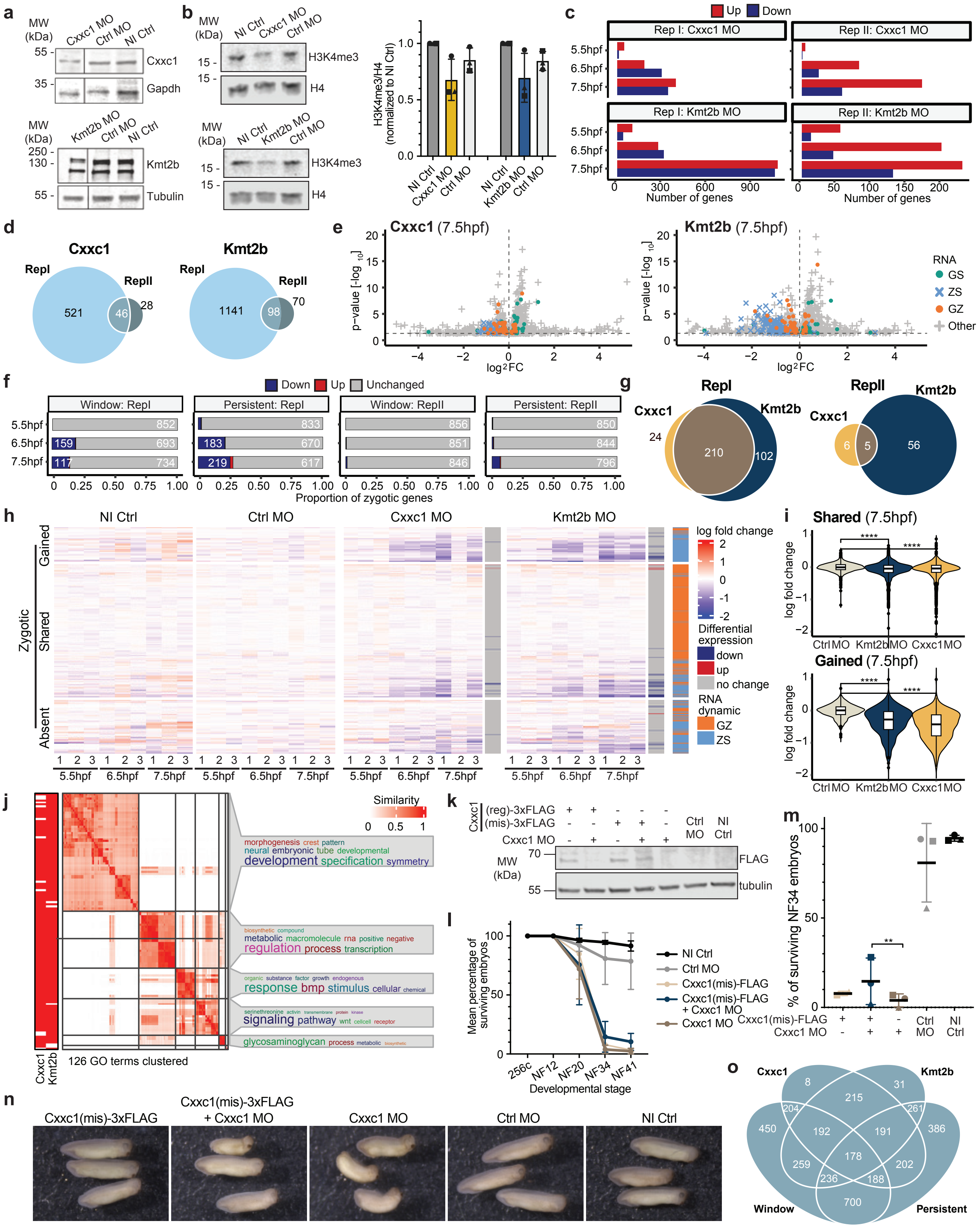

a
b
c
d
e
f
g
h
i
j
k
l
m
n
o
H3K4me3
H4
Kmt2b
Tubulin
Cxxc1
Gapdh
Rep I: Cxxc1 MO
Rep II: Cxxc1 MO
Rep I: Kmt2b MO
Rep II: Kmt2b MO
Number of genes
Proportion of zygotic genes
126 GO terms clustered
Developmental stage
% of surviving NF34 embryos
Window
Persistent

Figure S7. Cxxc1 and Kmt2b are required for proper ZGA and embryonic development.
(a) Western blots showing Cxxc1 and Kmt2b protein levels in Cxxc1 and Kmt2b knockdown embryos. (b) (Left) Western blot showing H3K4me3 levels in nuclear fractions of Cxxc1 and Kmt2b knockdown embryos compared to non-injected control and control morpholino-injected embryos at NF12. (Right) Quantification of H3K4me3 signal intensity relative to H4 for each condition. (c) Counts of differentially expressed genes at 5.5hpf, 6.5hpf and 7.5hpf time points in Cxxc1 and Kmt2b morpholino-injected conditions compared to control morpholino embryos (adj. p-value < 0.05) for both biological replicates. (d) Overlap of downregulated genes in Cxxc1 knockdown (top) and Kmt2b knockdown (bottom) conditions compared to control morpholino across all three timepoints between both biological replicates. (e) Volcano plots displaying expression fold change and adj. p-value (cutoff: 0.05) in knockdown conditions compared to control morpholino for biological replicate 2 at 7.5hpf colored by RNA dynamics groups. Density plots on top represent the distributions of $\log_2$ fold change for the RNA dynamics group. (f) Number of differentially expressed and unchanged zygotic genes at 5.5hpf, 6.5hpf and 7.5hpf time points in Cxxc1 and Kmt2b morpholino-injected conditions compared to control morpholino embryos in each biological replicate (adj. p-value < 0.05). *Zygotic genes = Gamete-Zygotic (GZ) + Zygotic-Specific (ZS).* (g) Overlap of downregulated zygotic genes across all three timepoints between Cxxc1 and Kmt2b morpholino conditions compared to the control morpholino for biological replicate 1 (left) and replicate 2 (right). (h) Expression of zygotic genes in 3 technical replicates of every sample for biological replicate 2, clustered by H3K4me3 dynamics group (SHARED, GAINED and ABSENT). Log_2 fold change of every gene is calculated over the mean TPM of three technical replicates of control morpholino of the respective time point. Genes are annotated with differential expression status separately for the Cxxc1 and Kmt2b knockdown conditions, as well as by RNA dynamics group. (i) Log_2 fold change of gene expression levels of all zygotic genes calculated over mean TPM of control morpholino technical replicates for biological replicate 2 for SHARED (top) and GAINED (bottom) groups at 7.5hpf. Statistical test: one-sided Wilcoxon rank-sum test with alternative hypothesis that values of left distribution tend to be larger than right distribution; p-values: (****)<=0.0001, (***)<=0.001, (**) <= 0.01, (*)<=0.05; n.s. are p-values > 0.05. (j) GO analysis of downregulated zygotic genes from Cxxc1 and Kmt2b knockdown conditions across all timepoints. Significantly enriched GO terms (adj. p-value < 0.01) are clustered based on their similarity. (k) Western blot of cytoplasmic fractions from pre-ZGA embryos injected with 100pg 3xFLAG-tagged Cxxc1 mRNA tagged under different conditions. “Cxxc1(reg)-3xFLAG” denotes wild-type Cxxc1 mRNA; “Cxxc1(mis)-3xFLAG” contains a 5-base mismatch at the morpholino target site. Tubulin is used as a loading control. (l) Survival analysis of embryos injected with Cxxc1(mis)-FLAG mRNA with and without Cxxc1 morpholino (MO), compared to Cxxc1 MO alone, and Ctrl MO and non-injected controls. Line plot shows the percentage of embryos surviving across key developmental timepoints (X-axis). Y-axis indicates the mean percentage of embryos (calculated from pre-ZGA sorted embryos) reaching each stage. Data represent n=3 independent experiments, N>35 embryos per condition; error bars indicate standard deviation. (m) Column scatter plot showing the weighted mean percentage of surviving embryos at NF34 for each biological replicate (n=3). Independent biological replicates are represented with different point styles (n=3). Error bars represent standard deviation across replicates. Asterisks denote significance from Fisher’s exact test (one-tailed) performed on pooled embryo count data for each condition (alive vs dead embryos). **P-value < 0.01. (n) Representative phenotype images of embryos for each rescue experiment condition at NF34. (o) Venn diagram representing the overlap of downregulated genes across Cxxc1 knockdown, Kmt2b knockdown, window H3K4 methylation depletion and persistent H3K4 methylation depletion conditions (from Fig.5). Gene counts denote the union of significantly downregulated genes versus their controls (p value < 0.05) across all technical replicates of the respective experiment.

Table S1. Number of genes overlapping between each H3K4me3 dynamics group and RNA dynamics group

		RNA dynamics groups			
		Gamete-Specific	Gamete+ Zygotic	Zygotic-Specific	Not-detected
H3K4me3 dynamics groups	LOST	122	1	0	585
	SHARED	320	377	96	1112
	GAINED	0	16	105	382
	ABSENT	329	49	140	12100

Supplementary methods

Immunofluorescence and Image Analysis

Briefly, embryos were developed until the desired mid-ZGA stage (i.e. 6.5hpf/1000-cell stage) and fixed using 4% paraformaldehyde (PFA) in 1x MEM (100mM MOPS pH 7.4, 2mM EGTA, 1mM $MgSO_4$) overnight at 4°C, permeabilized in 100% methanol and stored at -20°C until further processing. Embryos were bleached in a solution of 4% formamide and 2% hydrogen peroxide in a 0.5× saline sodium citrate (SSC; 75 mM NaCl, 7.5 mM sodium citrate) buffer for approximately 4-6 hours, until evenly white. 5-EU-injected embryos were incubated with 100mM Tris-HCl, 1mM $CuSO_4$, Alexa Fluor 594 Azide (Thermo Fisher Scientific, #A10270) and 100mM ascorbic acid for 6 hours at room temperature in the dark to stain nascent RNA. Embryos were blocked in 1% BSA in TBST (1x TBS containing 0.1% Triton-X-100, pH 7.6) for 3 hours at RT, followed by primary antibody staining depending at 4°C overnight, with the following antibodies depending on the experiment: α-H4 (1:500; Abcam, ab81380), α-H3K4me3 (1:300; Abcam, ab8580). Secondary antibody incubation and DNA staining was simultaneously performed overnight at 4°C in the dark, with the following antibody fluorescent conjugates or stains depending on the experiment: goat α-mouse AF488 (1:500; Invitrogen, #A-11001), goat α-rabbit AF647 (1:500; Invitrogen, #A-21244), SiR-DNA (1:5000; Tebubio, #SC007). After staining, embryos were post-fixed in 4% PFA in 1x MEM for 2 hours at room temperature, dehydrated in methanol, and cleared in freshly made benzyl alcohol:benzyl benzoate (1:2) solution for 24–48 hours before imaging. All intermediate washes were performed as described earlier17,43. Cleared embryos were mounted in chambers constructed from cover slips and double-sided tape, filled with clearing reagent. Imaging was performed using a Nikon Ti-2 microscope equipped with Dragonfly confocal unit with a 20× Plan Apo NA 0.75 objective (Nikon) and a Zyla sCMOS camera (Andor), capturing z-stacks with 5 µm intervals and tiling with 2% overlap. Embryos were imaged from both sides to ensure complete signal capture.

For nuclear segmentation, a custom Cellpose model was trained by random extraction of optical sections from z-stacks using a Fiji macro (ImageJ version 1.54f) followed by manual annotation. Images were then split into training and test datasets and the Cellpose model "cyto3" was used for re-training with a mean diameter of 25 pixels determined from the average size of training masks. The training process lasted 300 epochs (Cellpose version 3.0.8, Python version 3.10.0, Python libraries Pytorch 2.3.1, CUDA 11.8, and cuDNN 8.7). The custom-trained model was used for nuclear segmentation on the respective DNA-staining channel, depending on the experiment. Over-segmented labels were corrected by enlarging objects in the images using the morphological operation "dilation". Identified masks were applied to other channels to measure intensity within the mask area and the "regionprops" module from the scikit-image library (version 0.23.2) was used to extract a set of parameters as the intensity per volume from the nuclei masks and the raw intensity images. Statistical analysis as well as plotting was performed in R (Version 4.3.1) with the packages ggplot2 (version 3.3.6), ggpubr (Version 0.6.0) and multcomp (Version 1.4-26). Figures for microscopy images were made in ImageJ (Version 1.54f). The analysis pipeline was run on an Intel i9 12900KF 24-Core Processor with 64 GB of RAM and an Nvidia RTX A4500 GPU (driver version 525.78.01).

MBD-seq sample collection, library preparation and sequencing

250x pre-ZGA (256-cell stage i.e. 4.5 hpf) embryos were collected per replicate and prepared using the chromatin-bound protein sample preparation method. The pellet was resuspended in 200µl 1x TE buffer (10 mM Tris-HCl pH 8, 0.1 mM EDTA) and incubated with 4µl RNase A (Sigma Aldrich, #10109142001) for 1 hour at 37°C. Samples were incubated for 1 hour with 10mg/ml proteinase K (Sigma Aldrich, #P6556) at 65°C, followed by heat-induced proteinase K inactivation by a 15 minute incubation at 95°C. 400µl of phenol/chloroform/isoamyl alcohol (25:24:1, pH 8) was added to the sample and vortexed for 30 seconds, followed by a 3 minute centrifugation at maximum speed for 3 minutes and upper, aqueous layer was collected. This step was repeated once. 100µl chloroform was added, sample was vortexed and centrifuged with the same conditions. Upper phase was transferred to a new tube, taking care to avoid chloroform contamination. Ethanol precipitation was performed by adding 12 µL glycogen (5 mg/mL), 40 µL sodium acetate (3 M, pH 5.2), and 1200 µL ethanol (96%), followed by overnight incubation at -20°C. The sample was centrifuged at 4°C for 10 minutes at maximum speed, followed by two 70% ethanol washes. Pellet was air-dried and eluted in 30µl nuclease-free water. Extracted DNA samples were sonicated with Bioruptor Pico (Diagenode) in 0.1ml microtubes (#C30010015) for 8 cycles (ON: 15s/OFF: 90s). Sonication to ~400bp fragment length was confirmed on a 0.8% agarose gel. Sample concentration was measured using Qubit and diluted using 1x TE buffer to a final concentration of 100 ng/nl. 1.2µg DNA was used for further processing, of which 10% was kept aside as input. Methylated DNA was captured and eluted from remaining samples in one single elution with 150µl High Elution Buffer using the Methylated DNA Capture kit (Diagenode: #C02020010) as described in their protocol. Samples were then purified using phenol:chloroform:isoamyl alcohol isolation and precipitated overnight at -20°C as described above. Samples were finally eluted in 50µl nuclease-free water and per sample, 10-20 ng of total DNA was processed for library preparation.

Sequencing libraries were generated using the NEBNext Ultra II DNA Library Prep Kit for Illumina (NEB, #E7645S) as per the manufacturer's instructions using 6-8 PCR amplification cycles. The quality of cDNA libraries was assessed using the Agilent High Sensitivity D5000 ScreenTape System (Agilent, #5067-5592) in the Agilent 4150 TapeStation System. Libraries were multiplexed and sequenced was performed as paired-end 50 bp reads on the Illumina NextSeq 1000 by the Helmholtz Core Facility Genomics (CF-GEN).

H3K4me3 dynamics group definitions

Broad peaks were called on publicly available spermatid, sperm, pre-ZGA and ZGA stage H3K4me3 ChIP-seq datasets12,39,40 as described in the ChIP-seq processing section above. H3K4me3 dynamics groups were defined based on the detection of a H3K4me3 peak within the gene promoter regions (TSS +/- 1kb) at each time point. Genes for which a H3K4me3 promoter peak is observed at all four time points are denoted as the "SHARED" group of genes. Genes for which a H3K4me3 peak is not detected in the spermatid stage, but is acquired during one of the subsequent stages are labelled as the "GAINED" group of genes. Similarly, genes for which the promoter H3K4me3 peak is detected at the spermatid stage and no longer detected from one of the subsequent stages are called the "LOST" group of genes. Genes for which we did not detect a H3K4me3 peak at any of the four time points were labelled as the "ABSENT" group of genes. These major H3K4me3 dynamics groups can be further categorized based on the timing at which the H3K4me3 peak is lost or gained i.e., the "LOST" group is subdivided into "Lost at sperm", "Lost at pre-ZGA" and "Lost at ZGA" and the "GAINED" group is subdivided into "Gained at pre-ZGA" and "Gained at ZGA". Genes that oscillate between loss and gain of H3K4me3 peaks at their promoters during the four time points were removed from further analysis. Additionally, we

removed all genes that gained a new peak at the promoter between the spermatid and sperm stages. Collection of spermatid and sperm chromatin samples involves their careful separation from neighbouring blood vessels using testes tissue homogenization and centrifugation, risking blood cell contamination. Indeed, gene ontology analysis of genes that gain new H3K4me3 promoter peaks in the sperm stage showed enrichment for blood and circulation related terms, suggesting possible contamination of blood DNA in the samples12.

Gene expression group definitions

RNA dynamics groups were defined with the help of published total RNA-seq datasets from spermatid40 and egg39 stages as well as published nascent RNA-seq datasets for embryos at 5hpf, 6hpf, 7hpf, 8hpf and 9hpf43. Alignment of sequencing reads and quantification of transcript abundance was performed as described in the "RNA-seq data processing" section below. First, nascent transcription at each time point (6/7/8/9hpf) was calculated based on the net increase of reads from the "background" pulldown at 5hpf, as described in the reference study43. Then, transcripts that could be detected above the defined threshold (TPM increase > 5) in all replicates of any embryonic time point (6/7/8/9hpf) were identified as zygotically expressed genes. This stringent list was split into two groups: (1) the genes that were also detected at the spermatid or egg stages were defined as the "Gamete+Zygotic" group (2) the remaining genes were defined as the "Zygotic-Specific" group. In order to define a strict "Gamete-Specific" group, we identified transcripts that satisfied the following conditions: (1) detected above the threshold (> 5 TPM) in the spermatid and/or egg stages, (2) not detected above the threshold (<= 0 TPM increase) in any replicate of the embryonic time points (6/7/8/9hpf). The "Not Detected" group is defined by all genes that are not detected above the threshold (<= 0 TPM increase) in any stage (spermatid, egg, 6/7/8/9hpf). As these gene groups are stringently filtered and are not exhaustive, all of the genes not included in the groups described above were excluded from further analysis in our study.

ZGA timing groups were defined by the first timepoint that a gene is detected above a threshold of 5 TPM in the nascent dataset in any replicate. Genes that are never detected above 5 TPM in any replicate are labeled as Non-Zygotic.

mRNA production

Four independent constructs: mouse Kdm5b (accession number NM_152895, aa1-770), its catalytic inactive (ci) mutant (H499A; aa1-770), *Xenopus* H3.3 (accession number NM_001098432) and its dominant-negative mutant (K4M) were sub-cloned into pCS2+ vector with *Xenopus* globin 5' and 3' UTRs followed by a polyadenylation signal-sequence of Simian Virus 40 (SV40), a C-terminus 3xHA tag and a C-terminus NLS-tag[13]. Additionally, the AID sequence from the pRN3P_AID-Dam-only construct (a kind gift by Maria-Elena Torres-Padilla; Addgene plasmid #136065)[73] was subcloned into each of the four plasmids between the protein sequence and 3x HA tag using Gibson assembly (Table 1). For auxin-induced protein degradation, the pRN3P-TIR1-3×Myc construct (a kind gift from Maria-Elena Torres-Padilla; Addgene plasmid #119766)[73] was used. mRNA for all five Kpn1-linearized constructs was synthesized from *in vitro* using the RiboMAX™ Large Scale SP6 RNA polymerase kit (Promega, #P1280) or the mMESSAGE mMACHINE T3 transcription kit (Thermo Fisher Scientific, #AM1348).

For Cxxc1 morpholino rescue experiments, full-length *Xenopus* Cxxc1.S was subcloned into pCS2+ vector with *Xenopus* globin 5' and 3' UTRs followed by a polyadenylation signal-sequence of Simian Virus 40 (SV40) and a C-terminus 3xFLAG tag. For the morpholino-resistant construct, a five base pair mismatch primer sequencing at the translation start site was used for PCR amplification prior to ligation using Gibson assembly (primer sequences are listed in Table 1). After linearization using restriction enzyme KpnI, mRNA was synthesized in vitro using the mMESSAGE mMACHINE SP6 transcription kit (Thermo Fisher Scientific, # AM1340).

CUT&RUN

45 whole embryos were collected per sample at mid-ZGA stage (6.5hpf). Embryos were first incubated in 1mg/ml pronase (Serva, 33635) diluted in 0.1x MMR for 5 minutes to digest the vitelline membrane. Embryo tissue was dissociated by incubation in Newport buffer 2.0[78] (0.1M sodium isethionate, 20mM sodium pyrophosphate, 10mM CAPS, pH to 10.5 using NaOH), followed by washes and resuspension in ice-cold nuclear extraction buffer (20mM HEPES-KOH, pH 7.9, 10mM KCl, 500 µM spermidine, 0.1% Triton X-100, 20% glycerol). Each sample was incubated with 150µl concanavalin A beads (Epicypher, 21- 1401) first washed with and then resuspended in 300µl binding buffer (20 mM HEPES-KOH pH 7.9, 10 mM KCl, 1mM CaCl2, 1mM MnCl2). Extracted nuclei were incubated in the bead suspension for 10 minutes at room temperature while rotating to allow their binding. The supernatant was then discarded, nuclei were blocked for 5 minutes at RT with blocking buffer (20 mM HEPES-KOH pH 7.5, 150 mM NaCl, 0.5 mM spermidine, 0.1% BSA, 2 mM EDTA,1x protease inhibitor) and then incubated in 1:100 primary antibody solution overnight at 4°C. After two washes with wash buffer (20 mM HEPES-KOH pH 7.5, 150 mM NaCl, 0.5 mM spermidine, 0.1% BSA, 1x protease inhibitor), nuclei were suspended in wash buffer and incubated with pAG-MNase (Epicypher, 15-1116) for 1 hour at 4°C, then washed twice again with wash buffer. MNase digestion was activated for exactly 30 minutes at 4°C by the addition of 3µl 100mM CaCl2, followed by quenching of the reaction with the addition of 150µl 2x STOP buffer (200 mM NaCl, 20 mM EDTA, 4 mM EGTA, 50 µg/mL RNase A, 40 µg/mL glycogen), containing 2ng exogenous spike-in DNA (E. coli, Epicypher 18-1401). Samples were then incubated at 37°C for 20 minutes to release chromatin fragments, following centrifugation at 16,000g for 5 min at 4°C. The supernatant containing soluble chromatin fragments was mixed with SDS and proteinase K (Sigma-Aldrich) at 70°C for 10 min and subjected to on-column DNA purification (QIAGEN, MiniElute PCR Purification Kit).

CUT&RUN sequencing libraries were prepared using NEB Ultra II DNA library prep kit (NEB, #E7645), following the manufacturer's instructions, using 12 PCR amplification cycles with CUT&RUN-specific cycling parameters (45s 98°C for polymerase activation, 14 cycles of 15s 98°C DNA melting and 10s 60°C primer annealing and short extension, 1 min 72°C final extension), without size selection. The libraries were sequenced on a NextSeq 1000 sequencing platform.

Total RNA extraction and ribosomal depletion

Three embryos were collected per sample and stored at -80°C. Total RNA was isolated using the RNeasy kit (Qiagen, #74106) according to the manufacturer's instructions. First, embryos were lysed in the RLT buffer by vortexing for 15 minutes at 4°C. DNase digestion was performed according to the manufacturer's instructions using RNase-free DNase (Qiagen, #79254). RNA

was eluted in 40µl nuclease-free H2O. Concentration was measured using Nanodrop. Per sample, 500ng of total RNA was used for ribosomal RNA depletion. Ribosomal RNA was depleted using custom-made oligomer mixes for Xenopus laevis rRNA as described previously79 with minor modifications. Briefly, RNA was hybridized with 1 µL each of probe mix 1 and probe mix 2 in 20 µL hybridization buffer (100mM Tris–HCl pH 7.4, 200mM NaCl, 10mM DTT) by incubating at 95°C for 2 minutes. Hybridized samples were treated with 10 units of RNase H (NEB, #M0523) at 65°C for 5 minutes, followed by 2 units of RQ1 RNase-free DNase (Promega, #M6101) at 37°C for 30 minutes.

Supplementary Table 2. Primer sequences used in Gibson assembly cloning.

**Name of primer**	**Primer sequence for cloning: 5' (overlap/spacer/ANNEAL) 3'**
AID forward	aagcaaagatcaagggtggg*cgcgcc*GGCAGTGTCGAGCTGAATC
AID reverse	gtagcttccaccgcctccgc*tcgagac*AGCTCTGCTCTTGCACTTC
H3.3 K4M forward	gccgcgccaccatgggccgg*cct*ATGGCCCGTACAATGCAGAC
H3.3 common reverse	attcagctcgacactgccgg*cgcgccc*AGCGCGCTCTCCGCGTAT
H3.3 wt forward	gccgcgccaccatgggccgg*cct*ATGGCCCGTACAAAGCAGACC
Cxxc1 forward	gccgcgccaccatgggccggccaGAGATGGAGAGTGAGTTTTCAGACG
Cxxc1 forward (morpholino-resistant)	gccgcgccaccatgggccggccaGAGATGGA**A**AG**C**GA**A**TT**C**TC**C**GACG CTGACC
Cxxc1 reverse	tcaccgcctccaccgctcggcgcgccGCGCTCTGTGTGTGTGCG

Supplementary Table 3. Sequences of antisense morpholino oligonucleotides used for t he knockdown experiments.

Name of asMO	asMO sequence
Kmt2b Oligo	5'-CATCTTTATCCGCTCTGGGAACTAC-3'
Cxxc1 Oligo	5'-AGCGTCTGAAAACTCACTCTCCATC-3'
Standard Control Oligo	5'-CCTCTTACCTCAGTTACAATTTATA-3'

Supplementary Table 4. Key resources used in this study

Reagent or resource	Source	Identifier, Lot number
**Primary antibodies**		
H3K4me3	Abcam	ab8580, 1041609-1
H3K4me1	Abcam	ab8859, 1040931-5
H3K9ac	Millipore	#6-942, 3286483
H3K27ac	Cell Signalling Technologies	#8173
H3K36me3	Abcam	ab9050, GR288636-2
H3K79me3	Diagenode	C15410068, A-86-0023P
H4	Abcam	ab31830, GR3401688-2
H3	Abcam	ab10799, 1084399-8
HA	Sigma-Aldrich	H9658, 128M4789V
Cfp1	Biomol	A303-161A
Kmt2b/MLL2	Abcam	ab104444, GR67027-52
IgG	Cell Signalling Technologies	2729S, lot 10
FLAG	Sigma	F1804
**Secondary antibodies**		
IRDye 680LT (donkey anti-mouse)	LICOR	926-68022
IRDye 800CW (donkey anti-rabbit)	LICOR	926-32213
**Kits**		
Methylated DNA Capture kit	Diagenode	C02020010
NEBNext Ultra II Directional RNA Library Prep Kit for Illumina	NEB	E7760L
NEBNext Multiplex Oligos for Illumina Set 1/2/3 (96 Unique Dual Index Primer Pairs)	NEB	E6440S/E6442S/E6444S

Software	Version	
GraphPad Prism	10	
IGV	2.13.0	
Fiji ImageJ	2.14.0/1.54f	
ImageStudio	5.5.4	
SnapGene Viewer	7.1.1	
