# Supplementary material for: Pre-marking chromatin with H3K4 methylation is required for accurate zygotic genome activation and development
Source: Nat Commun. 2025 Dec 19;16:11334. doi: 10.1038/s41467-025-67692-7 (PMC12722762; doi:10.1038/s41467-025-67692-7)
Supplement: Supplementary file 2 — Reporting Summary [file 41467_2025_67692_MOESM2_ESM.pdf]

nature portfolio

Corresponding author(s): Eva Hörmanseder, Meghana S. Oak

Last updated by author(s): Nov 15, 2025

Reporting Summary

Nature Portfolio wishes to improve the reproducibility of the work that we publish. This form provides structure for consistency and transparency in reporting. For further information on Nature Portfolio policies, see our Editorial Policies and the Editorial Policy Checklist.

Statistics

For all statistical analyses, confirm that the following items are present in the figure legend, table legend, main text, or Methods section.

n/a	Confirmed	
☐	☒	The exact sample size (*n*) for each experimental group/condition, given as a discrete number and unit of measurement
☐	☒	A statement on whether measurements were taken from distinct samples or whether the same sample was measured repeatedly
☐	☒	The statistical test(s) used AND whether they are one- or two-sided<br>*Only common tests should be described solely by name; describe more complex techniques in the Methods section.*
☐	☒	A description of all covariates tested
☐	☒	A description of any assumptions or corrections, such as tests of normality and adjustment for multiple comparisons
☐	☒	A full description of the statistical parameters including central tendency (e.g. means) or other basic estimates (e.g. regression coefficient) AND variation (e.g. standard deviation) or associated estimates of uncertainty (e.g. confidence intervals)
☐	☒	For null hypothesis testing, the test statistic (e.g. *F*, *t*, *r*) with confidence intervals, effect sizes, degrees of freedom and *P* value noted<br>*Give P values as exact values whenever suitable.*
☒	☐	For Bayesian analysis, information on the choice of priors and Markov chain Monte Carlo settings
☒	☐	For hierarchical and complex designs, identification of the appropriate level for tests and full reporting of outcomes
☒	☐	Estimates of effect sizes (e.g. Cohen's *d*, Pearson's *r*), indicating how they were calculated

Our web collection on statistics for biologists contains articles on many of the points above.

Software and code

Policy information about availability of computer code

Data collection	No software was used for data collection.
Data analysis	Histone tail mass spectrometry data analysis was performed with the Skyline (version 21.2).<br>Immunofluorescence and Image Analysis: For nuclear segmentation, a custom Cellpose model was trained by random extraction of optical sections from z-stacks using a Fiji macro (ImageJ version 1.54f) followed by manual annotation. Images were then split into training and test datasets and the Cellpose model "cyto3" was used for re-training with a mean diameter of 25 pixels determined from the average size of training masks. The training process lasted 300 epochs (Cellpose version 3.0.8, Python version 3.10.0, Python libraries Pytorch 2.3.1, CUDA 11.8, and cuDNN 8.7). The custom-trained model was used for nuclear segmentation on the respective DNA-staining channel, depending on the experiment. Over-segmented labels were corrected by enlarging objects in the images using the morphological operation "dilation". Identified masks were applied to other channels to measure intensity within the mask area and the "regionprops" module from the scikit-image library (version 0.23.2) was used to extract a set of parameters as the intensity per volume from the nuclei masks and the raw intensity images. Statistical analysis as well as plotting was performed in R (Version 4.3.1) with the packages ggplot2 (version 3.3.6), ggpubr (Version 0.6.0) and multcomp (Version 1.4-26). Figures for microscopy images were made in ImageJ (Version 1.54f). The analysis pipeline was run on an Intel i9 12900KF 24-Core Processor with 64 GB of RAM and an Nvidia RTX A4500 GPU (driver version 525.78.01).<br>MBD-Seq and ChIP-Seq: Sequencing reads were aligned to the Xenopus laevis genome (v10.1) with bowtie (v4.2). The alignment process excluded discordant and mixed reads, with paired-end reads mapped to the reference genome. Aligned reads were converted to BAM format and filtered to retain only properly paired alignments. HOMER (v4.11) was used for peak identification against corresponding input controls. Broad peaks were called using histone-style parameters, while narrow peaks were called with default settings. Identified peaks were converted to BED format for downstream analysis.<br>CATaDa data: Analysis of fastq-files from CATaDa experiments was performed with the damidseq pipeline script63. Preprocessed fastq-files were mapped to the Xl.v10.1 genome assembly using bowtie2, and mapped reads were binned into fragments delineated by 5'-GATC-3'

motifs. Files were converted to the bigwig file format with bedGrapgToBigWig (v4) for visualization with the Integrative Genomics Viewer (IGV) (v2.13.0). MACS (v3.0.1) was used to call broad peaks on the mapped read (.bam) files generated by the damidseq_pipeline. The resulting coverages from the preprocessings explained above were log2 transformed after adding a pseudo count of 1. Final signal intensities for Meta plots and Heatmaps were calculated by adding up the coverage of all replicates. Aggregate line plots, depicting the mean signal intensity of gene sets of interest with bands representing standard error of the mean, were generated using ggplot2 (v3.4.4). Heatmaps were generated using the EnrichedHeatmap package (v1.32.0) in R.

All original code can be accessed at Zenodo (doi.org/10.5281/zenodo.17667122) or GitHub (https://github.com/ScialdoneLab/h3k4me3_maintenance). The preprocessed data for the analysis can be downloaded from Zenodo (https://doi.org/10.5281/zenodo.14648394).

Other SW used: GraphPad Prism 10, IGV 2.13.0, ImageStudio 5.5.4 ,SnapGene Viewer 7.1.1

For manuscripts utilizing custom algorithms or software that are central to the research but not yet described in published literature, software must be made available to editors and reviewers. We strongly encourage code deposition in a community repository (e.g. GitHub). See the Nature Portfolio guidelines for submitting code & software for further information.

Data

Policy information about availability of data

All manuscripts must include a data availability statement. This statement should provide the following information, where applicable:

- Accession codes, unique identifiers, or web links for publicly available datasets
- A description of any restrictions on data availability
- For clinical datasets or third party data, please ensure that the statement adheres to our policy

Original data produced in the experiments has been deposited on GEO. The MBD-seq data is available under the accession number GSE286525. The CATaDa data is available under the accession number GSE286524. The RNA-seq data from the auxin experiment is available under the accession number GSE286887. The CUT&RUN data is available under accession number GSE303651. The RNA-seq data from the morpholino treatments is available under the accession number GSE286886. The mass spectrometry proteomics data have been deposited to the ProteomeXchange Consortium via the PRIDE66 partner repository with the dataset identifier PXD058937. Publicly available data were obtained from GEO with the following accession numbers: GSE125982, GSE75164, GSE76059, GSE201835. All other relevant source data supporting the key findings of this study are provided with this paper. Source data are provided with this paper.

Research involving human participants, their data, or biological material

Policy information about studies with human participants or human data. See also policy information about sex, gender (identity/presentation), and sexual orientation and race, ethnicity and racism.

Reporting on sex and gender	*Use the terms sex (biological attribute) and gender (shaped by social and cultural circumstances) carefully in order to avoid confusing both terms. Indicate if findings apply to only one sex or gender; describe whether sex and gender were considered in study design; whether sex and/or gender was determined based on self-reporting or assigned and methods used.* *Provide in the source data disaggregated sex and gender data, where this information has been collected, and if consent has been obtained for sharing of individual-level data; provide overall numbers in this Reporting Summary. Please state if this information has not been collected.* *Report sex- and gender-based analyses where performed, justify reasons for lack of sex- and gender-based analysis.*
Reporting on race, ethnicity, or other socially relevant groupings	*Please specify the socially constructed or socially relevant categorization variable(s) used in your manuscript and explain why they were used. Please note that such variables should not be used as proxies for other socially constructed/relevant variables (for example, race or ethnicity should not be used as a proxy for socioeconomic status).* *Provide clear definitions of the relevant terms used, how they were provided (by the participants/respondents, the researchers, or third parties), and the method(s) used to classify people into the different categories (e.g. self-report, census or administrative data, social media data, etc.)* *Please provide details about how you controlled for confounding variables in your analyses.*
Population characteristics	*Describe the covariate-relevant population characteristics of the human research participants (e.g. age, genotypic information, past and current diagnosis and treatment categories). If you filled out the behavioural & social sciences study design questions and have nothing to add here, write "See above."*
Recruitment	*Describe how participants were recruited. Outline any potential self-selection bias or other biases that may be present and how these are likely to impact results.*
Ethics oversight	*Identify the organization(s) that approved the study protocol.*

Note that full information on the approval of the study protocol must also be provided in the manuscript.

Field-specific reporting

Please select the one below that is the best fit for your research. If you are not sure, read the appropriate sections before making your selection.

[x] Life sciences [ ] Behavioural & social sciences [ ] Ecological, evolutionary & environmental sciences

For a reference copy of the document with all sections, see nature.com/documents/nr-reporting-summary-flat.pdf

Life sciences study design

All studies must disclose on these points even when the disclosure is negative.

Sample size	Approximately 40 embryos were used as the sample size for each developmental assay, twice the recommended sample size as described in standardized development assay protocols for larval amphibian growth and development.
Data exclusions	No data was excluded from analysis.
Replication	All key experimental findings were replicated across multiple independent biological experiments. Mass spectrometry was performed on 3 independent biological replicates. MBD-seq and CATaDa experiments were performed on 2 independent biological replicates. RNA-seq experiments were performed with 3 independent technical replicates across 2 separate biological batches. Developmental outcome assays were replicated across 3 independent experiments comprising of minimum 35 embryos per condition per biological replicate. CUT&RUN experiments were performed on three biological replicates.
Randomization	Embryos were randomly assigned to experimental treatment groups.
Blinding	Blinding was not performed.

Reporting for specific materials, systems and methods

We require information from authors about some types of materials, experimental systems and methods used in many studies. Here, indicate whether each material, system or method listed is relevant to your study. If you are not sure if a list item applies to your research, read the appropriate section before selecting a response.

Materials & experimental systems

n/a	Involved in the study	
☐	☒	Antibodies
☒	☐	Eukaryotic cell lines
☒	☐	Palaeontology and archaeology
☐	☒	Animals and other organisms
☒	☐	Clinical data
☒	☐	Dual use research of concern
☒	☐	Plants

Methods

n/a	Involved in the study	
☒	☐	ChIP-seq
☒	☐	Flow cytometry
☒	☐	MRI-based neuroimaging

Antibodies

Antibodies used	H3K4me3 Abcam ab8580<br>H3K4me1 Abcam ab8859<br>H3K9ac Millipore #6-942<br>H3K27ac Cell Signalling Technologies #8173<br>H3K36me3 Abcam ab9050<br>H3K79me3 Diagenode C15410068<br>H4 Abcam ab31830<br>H3 Abcam 10799<br>HA Sigma H9658<br>Cfp1 Biomol A303-161A<br>Kmt2b/MLL2 Abcam ab104444<br>IgG Cell Signalling 2729S<br>FLAG Sigma F1804
Validation	Antibodies used in this study were commercially available and have been validated by the manufacturer. For Western Blot, immunofluorescence and CUT&RUN experiments, only antibodies suitable for this purpose were used, as specified by the manufacturer.

Animals and other research organisms

Policy information about studies involving animals; ARRIVE guidelines recommended for reporting animal research, and Sex and Gender in Research

Laboratory animals	Embryos of unknown sex were generated from adult Xenopus laevis males and females
Wild animals	This study did not involve wild animals.
Reporting on sex	Findings apply to embryos of unknown sex. Wherever applicable, samples derived from male/female animals have been specified. As

	explicitly noted in the manuscript, technical limitations in the field underlie the lack of sex-based equality in data inference.
Field-collected samples	This study did not involve samples collected from the field.
Ethics oversight	All frog maintenance and care were conducted according to the German Animal Welfare Act. Research animals were used following guidelines approved and licensed by ROB-55.2-2532.Vet_02-23-126.

Note that full information on the approval of the study protocol must also be provided in the manuscript.

Plants

Seed stocks	*Report on the source of all seed stocks or other plant material used. If applicable, state the seed stock centre and catalogue number. If plant specimens were collected from the field, describe the collection location, date and sampling procedures.*
Novel plant genotypes	*Describe the methods by which all novel plant genotypes were produced. This includes those generated by transgenic approaches, gene editing, chemical/radiation-based mutagenesis and hybridization. For transgenic lines, describe the transformation method, the number of independent lines analyzed and the generation upon which experiments were performed. For gene-edited lines, describe the editor used, the endogenous sequence targeted for editing, the targeting guide RNA sequence (if applicable) and how the editor was applied.*
Authentication	*Describe any authentication procedures for each seed stock used or novel genotype generated. Describe any experiments used to assess the effect of a mutation and, where applicable, how potential secondary effects (e.g. second site T-DNA insertions, mosiacism, off-target gene editing) were examined.*
